# Book Review: Physician's Field Guide to Neuropsychology: Collaboration Through Case Example

**DOI:** 10.3389/fpsyg.2021.548561

**Published:** 2021-02-16

**Authors:** Som Singh, Fahad Qureshi, Shipra Singh

**Affiliations:** ^1^Department of Biomedical Sciences, University of Missouri – Kansas City School of Medicine, Kansas City, MI, United States; ^2^QUICK Research Institute, Kansas City, MI, United States; ^3^Department of Psychiatry, Central Michigan University College of Medicine, Mount Pleasant, MI, United States

**Keywords:** neuropsychology, neurocognitive exam, psychology, interprofessional care, case studies

Interprofessional care between physicians and other healthcare professionals is key to successful patient care. The book *Physician's Field Guide to Neuropsychology: Collaboration through Case Example* is an excellent example of a foundational reading for physicians and other practitioners involved in neuropsychologic patient care. The book is aimed toward physicians who are not formally trained specialists in behavioral neurology or neuropsychiatry but desire the skills to be adept in related patient care while still aligning with the consensus statement set forth by the American Academy of Clinical Neuropsychology (Heilbronner et al., 2009). The book begins by discussing the importance of a patient's neurocognitive functioning, referred to as the “sixth vital sign” (Sanders, 2019). It does a good job of separating neuropsychology from its namesake branching fields. Moreover, the book outlines three major goals: (1) to emphasize that neuropsychological testing is completely different from cognitive screens and requires extensive training; (2) to clearly demonstrate the utility of neuropsychology through case examples in medical settings; and (3) to foster collaboration between neuropsychology and medical cultures to increase coordinated care (Sanders, 2019). These goals are well-resonated throughout the reading of this book.

The book is divided into three parts. The initial part contains chapters designed for the reader to develop an understanding of the clinical significance of neuropsychology. The second part involves case studies, and the third part involves interprofessional considerations. This organization allows for a reader to build their understanding as they use the book as well as hone their cognitions through practice questions at the end of chapters. The questions are well-designed, concise, and provide both providers and students with adequate assessment.

In evaluating the case studies provided, each case provides a standard layer composed of sections such as an introduction of what the case will focus on (i.e., pediatric traumatic brain injury, nuero-oncology, autism, sickle cell disease, etc.), followed by the case presentation, neurological exam findings, and other components which lead to the conclusion and collaborative discussion for the reader. Moreover, the organized approach of this book provides the reader with a smooth transition without requiring significant extraneous thought processing. This type of organization aligns with current literature recommendations of case study layouts (Meganck et al., 2017; Willemsen et al., 2017). In some regards, this book arguably can provide a consolidated neuropsychology training resource that some textbooks cannot provide.

The conscientious approach of this book is further seen in the third part regarding special considerations in neuropsychological evaluation. This portion separates this book from conventional textbooks, which aim to teach readers the primary material and forgo special considerations based on the assumption that medical practice will supplement this motion. Special considerations include a situation where the reading provider is likely to witness, such as if a medical interpreter is involved in care or evaluating present cultural factors of the patient.

Additionally, the interprofessional emphasis provided in this book aligns with prominent literature related to reducing medical error; of note are the feedback mechanisms (National Academies of Sciences, Engineering, and Medicine, 2015). This book contains a dedicated portion to patient feedback. The book details how to manage the situation as a provider and well as providing concise goals for the user to refer to. Interprofessional care allows for a reduction of human error, a key antagonist in patient care that can be avoided if properly trained.

The goal of *Physician's Field Guide to Neuropsychology: Collaboration through Case Example* is to provide non-specialized providers with proper support in neuropsychologic situations. There are noteworthy shortcomings seen in this piece of literature. It could have presented additional case studies, such as using neuropsychology in drug addiction, and the author could refer to research on such (Kong et al., 2018; Singh et al., 2019). Additionally, this review recommends this book to the audience of specialized providers as well if used as a supplementary review. Furthermore, if a reader desires to seek out further understanding of neuropsychology, this review recommends a secondary review of this book followed by potential formal textbook readings that students in training would use.

## Author Contributions

SoS oversaw the development of the project. FQ and ShS oversaw in the review and adaptation of this project. All authors listed have made a substantial, direct and intellectual contribution to the work, and approved it for publication.

## Conflict of Interest

The authors declare that the research was conducted in the absence of any commercial or financial relationships that could be construed as a potential conflict of interest.
